# Next-generation UWB antennas gadgets for human health care using SAR

**DOI:** 10.1186/s13638-021-01906-6

**Published:** 2021-02-16

**Authors:** Aysha Maryam Ali, Mohammed A. Al Ghamdi, Muhammad Munwar Iqbal, Shehzad Khalid, Hamza Aldabbas, Saqib Saeed

**Affiliations:** 1 University of Engineering and Technology, Taxila, Pakistan; 2 Computer Science Department, Umm Al-Qura University, Makkah City, Saudi Arabia; 3 Computer Engineering Department, Bahria University, Islamabad, Pakistan; 4 Software Engineering Department, Prince Abdullah Bin Ghazi Faculty of Information and Communication Technology, Al-Balqa Applied University, Al-Salt, Jordan; 5 Department of Computer Information Systems, College of Computer Science and Information Technology, Imam Abdulrahman bin Faisal University, Dammam, Kingdom of Saudi Arabia

**Keywords:** Wireless body area networks, Signal processing, Patient monitoring, Healthcare applications, WSN, Smart applications, Signal analysis

## Abstract

The body area network is now the most challenging and most popular network for study and research. Communication about the body has undoubtedly taken its place due to a wide variety of applications in industry, health care, and everyday life in wireless network technologies. The body area network requires such smart antennas that can provide the best benefits and reduce interference with the same channel. The discovery of this type of antenna design is at the initiative of this research. In this work, to get a good variety, the emphasis is on examining different techniques. The ultra-wide band is designed, simulated, and manufactured because the ultra-wide band offers better performance compared to narrowband antennas. To analyze the specific absorption rate, we designed a multilayer model of human head and hand in the high-frequency structure simulator. In the final stage, we simulated our antennas designed with the head and hand model to calculate the results of the specific absorption rate. The analysis of the specific absorption rate for the head and hand was calculated by placing the antennas on the designed model.

## Introduction

The wide use of wireless networks and the constant miniaturization of electrical devices have enabled the development of wireless body area networks for the body. In these networks, numerous sensors are attached to clothing or the body or even embedded under the skin. The wireless nature of the network and the wide variety of sensors offer many new, practical, and innovative applications to improve health care and quality of life. In this research, we are working on the design on ultra-wide band (UWB) wearable antennas, analyzing their results, and calculating their specific absorption rate (SAR) to achieve the best results, many simulations are carried out on high-frequency structure simulator (HFSS), and the work is done in the laboratory to keep the proposed antenna on the human body in the stable channel and to get the best results [1]. UWB transmits signals from most obstacles that generally reflect signals with limited bandwidth and a higher map. Its signals range from 3.1 GHz to 10.6 GHz, with a higher degree of attenuation [2]. Human tissues are affected by the absorption of electromagnetic (EM) frequency fields. The SAR parameter mainly takes into account the amount of energy absorbed by these tissues [3, 4].

When human tissues are bare and exposed to the electromagnetic field, we can calculate its SAR, where SAR belongs to mass per block in power per unit. SAR is usually communicated in watts/kg [5, 6]. For the analysis and measurement of SAR, there is a need for advanced specialized laboratory test equipment.

The required hardware includes a box (or a phantom), a sensor, and a mobile radio frequency mobile phone holder. SAR is measured more frequently by measuring phantom (head measurement) and box technique (body measurement). Mobile phones are placed directly on the head, which gives all the power. Radio frequency signals are measured by placing antennas or sensors at different locations. The system analyzes the aggregate data and then converts the radio frequency level signal to the SAR, mainly in a complicated way. The complete results are calculated on all operating frequencies or required.

The maximum level recorded is considered the SAR value about the spectrum (head). The specific absorption rate depends on frequency, conductivity, permittivity, distance, dielectric value, and the properties of the antenna [7]. Phantom requires all these values to be assigned to different tissue types at all working frequencies, where the spectrum must be exposed.

Software and analytical calculations also measured the dielectric values of the muscles, fat, skin, and bones. The results with distance from body parts and cell phones are used. Some cell phones are designed in one way so that the space between the human body is usually between 15 and 25 mm, depending on the structure of the cell phone. The small and possible separation represents the small distance created by the battery [8, 9]. The design of antennas for portable technologies is challenging due to the absorption of EM waves in the human body. Body area network (BAN) plays a vital role in the study of such challenges. The human body consists of many channels. That includes the dynamic nature of the channel and the problems of user acceptance. Preferably, an antenna designed has a gain pattern to meet the requirements of different links and to address the body channels. Finally, the proposed antenna must be small and flexible for user satisfaction by keeping SAR levels low. Our work analyzes in detail the above requirements and suggests antennas that address these challenges.

We have designed a multilayered model of human head and hand in the high-frequency structure simulator. During the final phase, we simulated our designed antennas with the head and hand model to calculate specific absorption rate results. Specific absorption rate analysis for head and hand was calculated by placing the antennas on the designed model.

## Methods/experimental

### Wearable antennas

“The portable antenna can be defined as an antenna designed for a part of the clothing” [5]. It is also deleted from the name of the antenna that can be carried in the body. These antennas are usually formed using a lightweight substrate, which is usually any textile product. These antennas are perfect to be used for smart applications. Portable health surveillance systems integrated into the telemedicine system are innovative information technologies that can support early detection and prevent serious consequences [10].

Technologies move toward the future where user-defined information is available on-demand to meet antenna requirements, which must focus on the body. Many portable antennas are available on the market in various devices and components, namely smart watches and glasses. Portable antennas have become popular because they need a future. Portable antennas have essential applications in the medical and security fields. They can transmit and receive signals when used in different parts of the body. Wearable (portable) antennas have effects on the human body if the radiation range is higher than the radiation range determined by FSS (FCC) [11].

Al-Turjman et al. [12] propose a context-sensitive seamless identity provisioning (CSIP) framework for the IIoT. The proposed mechanism can achieve the main security objectives of the WMSN in a short time. Many efforts of the human body affect the performance of the antenna, energy absorption in the body, SAR rate of the nearby antenna, and spread inside and outside the body to use and participate in cellular networks [11]. Therefore, many tools are needed to study the channels in the body for wireless BANs. New design and analysis are also needed. The portable health surveillance system, which may consist of a sensor and antenna integrated into the telemedicine system, is capable of supporting the early detection of abnormal diseases and preventing their severe consequences [13, 14].

Al-Turjman et al. [15] reflect the status of Internet of Medical Things (IoMT) for health care, including research and development plans and applications. The implementation of IoMT in health care has grown exponentially worldwide, but still presents design challenges many technical issues. Al-Turjman et al. [16] propose a pricing framework for a cloud node in the IoT era, taking into account uncertainty factors such as network topology, transmission/reception energy, load, and power. Besides, the results show that in all test cases, the GA has a lower operating time than that of the SAA with 68% improvement in the centralized version and 66% improvement in the distributed version in case the size of the uncertainty list is 256.

Ahmed et al. [17] rigorously analyze SAR impact on the human body with different parameters. Qidwai et al. [18] propose a noninvasive and cost-efficient health monitoring system that is more secure and rapidly deployable. An identification algorithm intelligent with unique wearable data is embedded. Muzammal et al. [19] defined a framework for the security of IoT devices conceivably. A “ScreenStealer” application is developed and explored the weaknesses. Jabbar et al. [20] proposed a technique that is focused on a generalized solution for a network of heterogeneous IoT devices with semantic interoperability. Semantically annotated data are used to build a new semantic mediator layer. Resource description framework (RDF) is used to store the semantic information, and for retrieval information from RDF, Sparkle query language is used.

Wireless body area networks (WBAN) are required to expand human service frameworks to enable them to more successful management and disease recognition, and emergency response in return for well-being [21]. Security and privacy in e-health surveillance with WBAN are mostly key to those individuals who suffer from diseases. The diseases related to the heart and  mental illnesses are required continuous cognition, because the whole network of these  workouts requires more security. These articles present security and protection issues [22, 23].

The human body is a piece of the driver to Insulation Materials. When the antenna is working close to the human body, it is detuned due to mismatches in impedance; part of the available energy is reflected and absorbed in the body, resulting in high absorption-specific values of SAR [24]. The standard unit is watt per kilogram (W/kg) for SAR.

Specific absorption rate (SAR) can measure wave penetration in human body tissues. It can also be considered as an “absorbed rate” related to the electric fields at a point, i.e.,

1 $${\text{SAR}} = \frac{{\sigma |E|^{2} }}{p}\left( {\frac{{\text{W}}}{{{\text{kg}}}}} \right).$$

The SAR can also be defined as the time derivative of the incremental energy (d*W*) dissipated in an incremental mass (d*m*) in a volume (d*V*) of a given density (*ρ*), which is described by Eqs. 2 and 3.

2 $${\text{SAR}} = \frac{{\text{d}}}{{{\text{d}}t}}\left( {\frac{{{\text{d}}W}}{{{\text{d}}m}}} \right) \Rightarrow \frac{{\text{d}}}{{{\text{d}}t}}\left( {\frac{{{\text{d}}W}}{{p({\text{d}}V)}}} \right)$$

3 $${\text{SAR}} = \frac{1}{p}\left( {\frac{{{\text{d}}W}}{{{\text{d}}t}}} \right)$$

where d*W* is the energy absorbed, d*m* is the mass, and d*V* is the volume element.

### Antenna designs: planar inverted cone antenna (PICA)

**Fig. 1:**
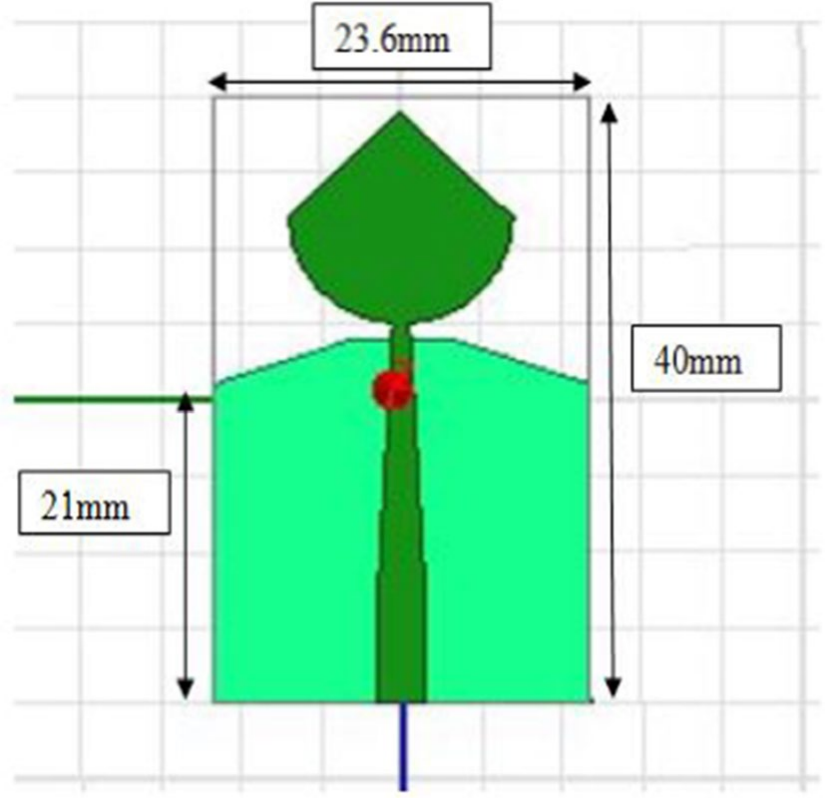
Micro-strip-line cone-shaped patch-fed. The substrate dimensions are 23.6 mm × 40 mm, a thickness is 1.6 mm, and a relative tolerance is 4.4. The PICA floor plan has dimensions of 23.6mmx23mm

We selected PICA for its impression of bandwidth efficiency as matched to its structural simplicity and small size unidirectional radiation pattern. The substrate is, in fact, a structure that retains the stain and serves as insulation between the connecting pieces. The substrate dimensions are 23.6 mm × 40 mm, a thickness is 1.6 mm, and a relative tolerance is 4.4. The PICA floor plan has dimensions of 23.6 mm × 23 mm, as shown in Fig. 1.

**Fig. 2:**
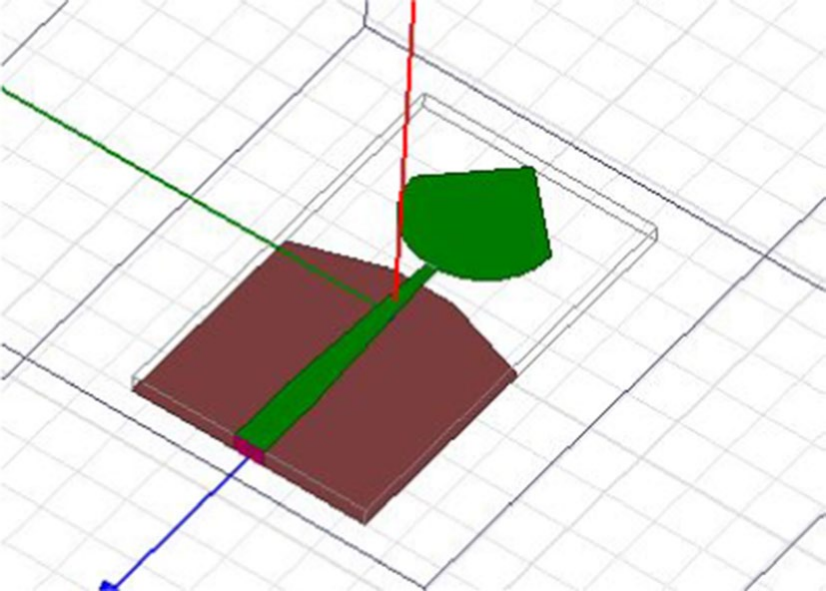
PICA antenna with the partial ground. A line is used to connect the two lines in order to match the calibration. The conical shape is fitted with a 50-μm threaded strip

PICA antenna with the partial ground is shown in Fig. 2. The conical shape is fitted with a 50-μm threaded strip. In order to match the calibration, we use a line to connect the two lines.

**Fig. 3:**
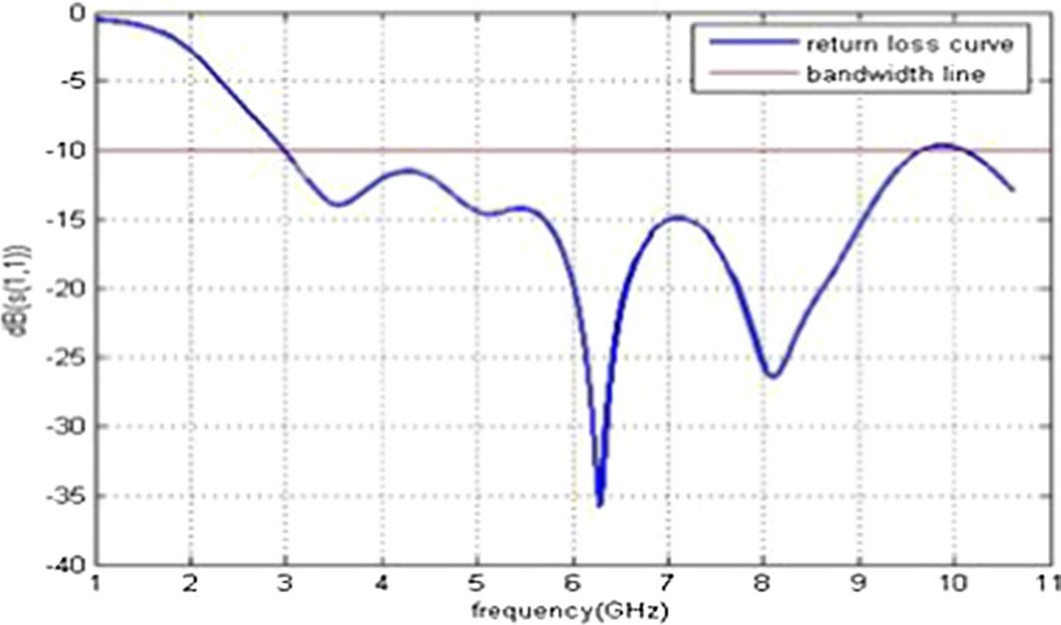
Return loss versus frequency. The return loss curve is 0 on the frequency 1 and -36 at the frequency of 6 GHz. Then, the frequency of return loss curve is increased and reached at -10 on the 10 GHz frequency

**Fig. 4:**
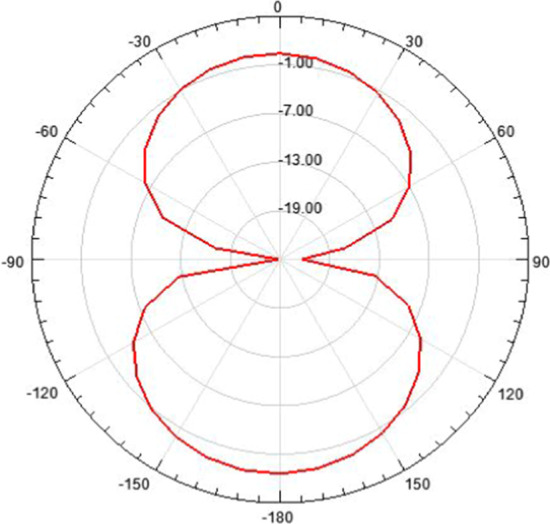
2.4 GHz radiation pattern. Antenna gain is a combination of directivity and electrical efficiency. Radiation pattern of the slotted multiband antenna at four different frequencies with major lobe

The simulations and results are shown in Fig. 3, where the return loss and frequency comparison are presented. Figure 4 presents the 2.4 GHz radiation pattern.

**Fig. 5:**
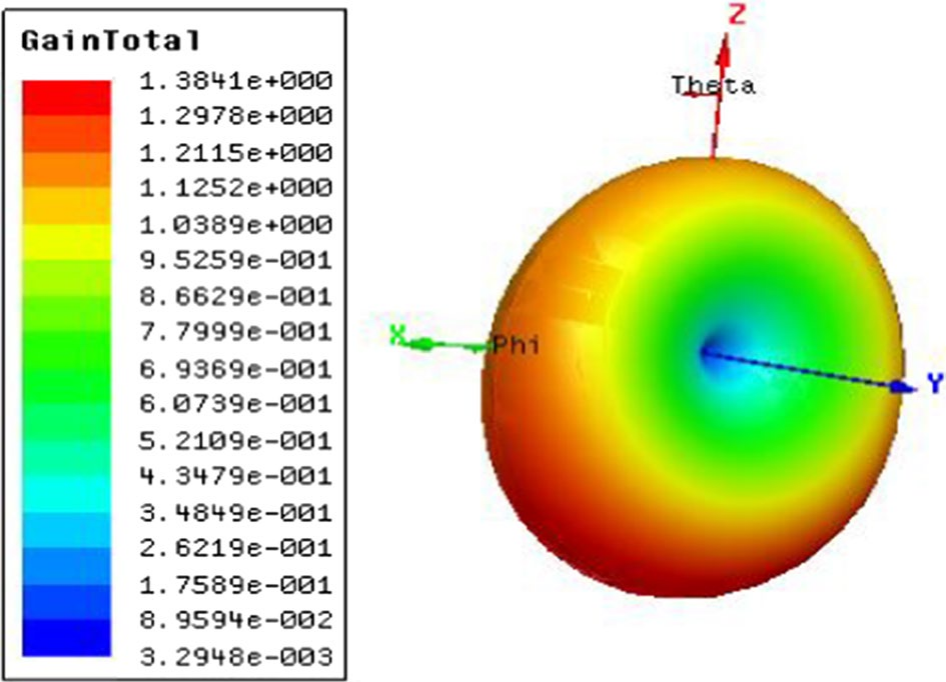
3D polar plot, which shows the 3D directions of x, y, and z, where x is the phi and z denotes the theta

The 3D polar plot is shown in Fig. 5, which demonstrates the *x*-, *y*-, and *z*-axes, where *x*-axis denotes the phi and *z*-axis denotes the theta.

Our results show that the PICA has a multidirectional radiation pattern, which makes the PICA antenna valuable for BAN applications. The width of the antenna beam indicates that the antenna has a high gain, as illustrated by the three-dimensional polarization scheme. The PICA high gain antenna is a demand for BAN applications. It is evident from the result that the gain is at 35.8185 dB, which makes antenna useful for analysis of SAR.

### Coplanar waveguide (CPW)-fed

PICA gives us better results for the UWB band, but the frequencies of mobile communications do not cover the bandwidth. While our study includes UWB communications and mobile communications, the CPW inverted planning cone is designed for mobile and UWB applications.

**Fig. 6:**
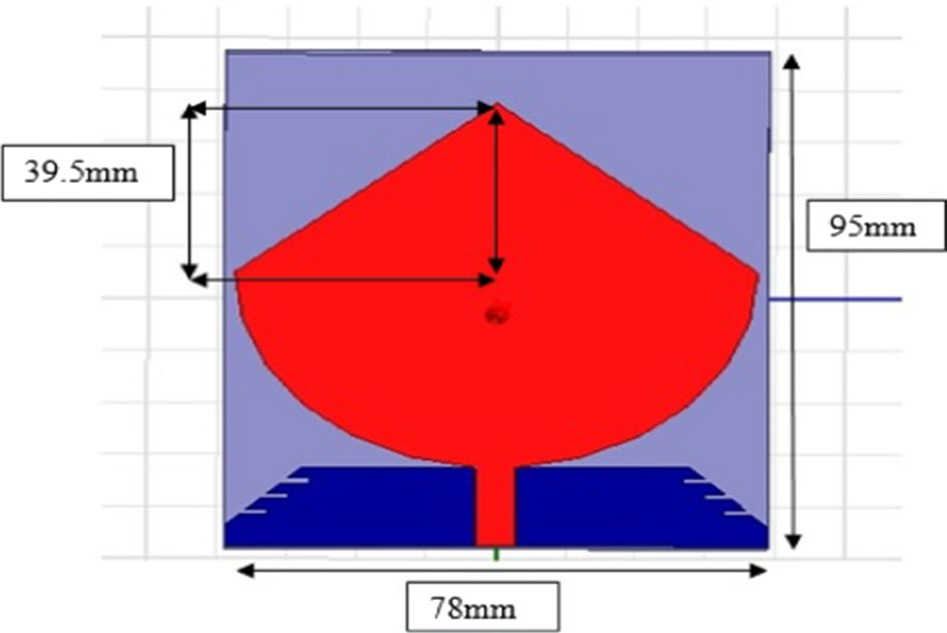
Cone antenna CPW-fed planer inverted. The antenna structure consists of mass, plate, and substrate. The rectangular disc is a radiating element cut into a cone supported by a conical, and an undulating ground plane

The antenna structure consists of a round plate, mass, and substrate; coplanar soil is used. The rectangular disc is a radiating element cut into a cone supported by a conical, and an undulating ground plane is shown in Fig. 6.

## Human head and hand design

### Human head model six layers

**Fig. 7:**
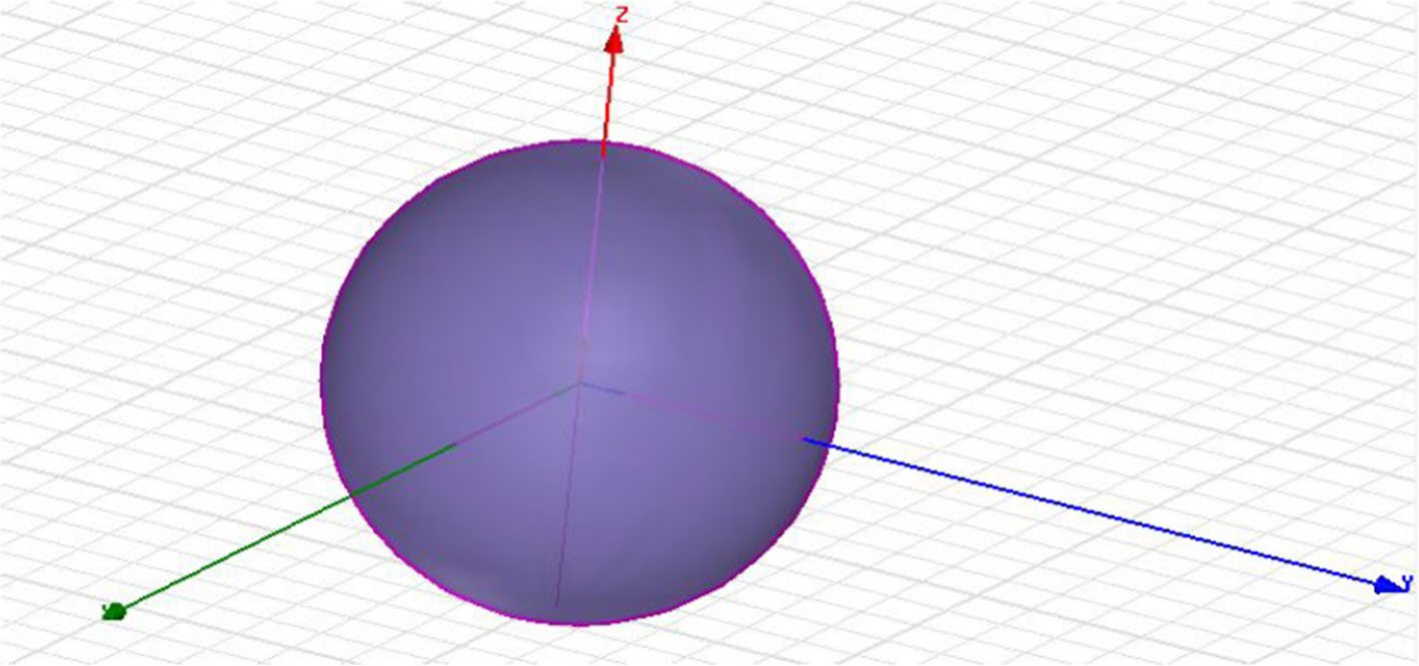
Head model in HFSS. Both bone and Dura have the same conductivity and permittivity values; only they differ in thickness. CSF makes the fifth layer and the brain is the last layer of our human head model

**Fig. 8:**
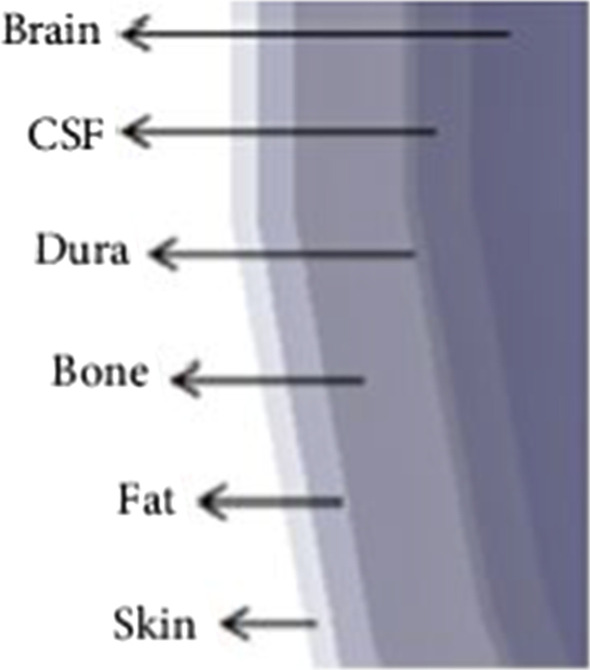
The human head model six layers are presented in here, where skin is at the initial level and brain is at the top

The first model consists of six layers, which differ in their conductivity and their relative permittivity. We used a scale model to design a human head in HFSS. The calculations are based on the head of an 18-year-old human. The outer layer is exposed to the EM field. The calculated values may differ from the actual values due to the presence of human hair on the outer layer, because the electrical properties of hair are different from those of human skin. The second layer is made of fat. We have varied the thickness of the external skin fat because the permittivity and conductivity values are low compared to the external layer. The third layer is made of bone, and the fourth layer is the Dura sphere. Bone and Dura have the same conductivity and permittivity values; only they differ in thickness. The LCR is the fifth layer and the brain is the last layer of our main human model, as shown in Figs. 7 and 8.

They both have the same conduction and permittivity values and vary in thickness. The main model and conductivity layers are presented in Table 1.

**Table 1:** Layers of head model and conductivity

Layers number	Conductivity (s/m)	Permittivity	Thickness (mm)
1. Skin	0.65	40.7	1
2. Fat	0.17	10	0.14
3. Bone	0.33	20.9	0.41
4. Dura	0.65	40.7	0.5
5. CSF	2.14	79.1	0.2
6. Brain	2.14	79.1	81

### Eight-layer hand model

**Fig. 9:**
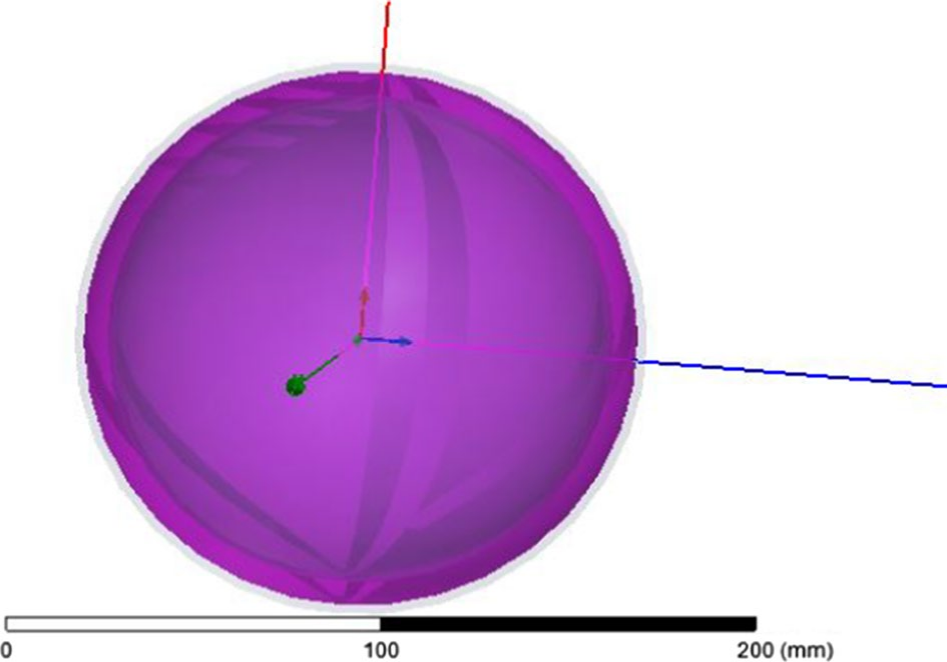
Head model (bowl model). Eight layers are presented in this figure, which starts from 0 and ended at 200 mm

The human head is a multilayered substance. That consists of *x*-, *y*-, and *z*-axes. For more accurate results, we need to add more levels to our model as shown in Fig. 9. Thus, we add two more layers to our model as shown in Table 2. The next layer added is a grey matter which is a part of our brain and contains neurons. Also, white matter is added, which also contains cells. By adding these two layers, our SAR analysis is more valuable to study the effect of EM waves on the human head.

**Table 2:** Seventh and eighth layers of head model and conductivity

Layer number	Conductivity (s/m)	Permittivity	Thickness (mm)
7. Grey matter	1.02	62.71	2.3
8. White matter	0.9	47.5	1.7

### Human hand model

**Fig. 10:**
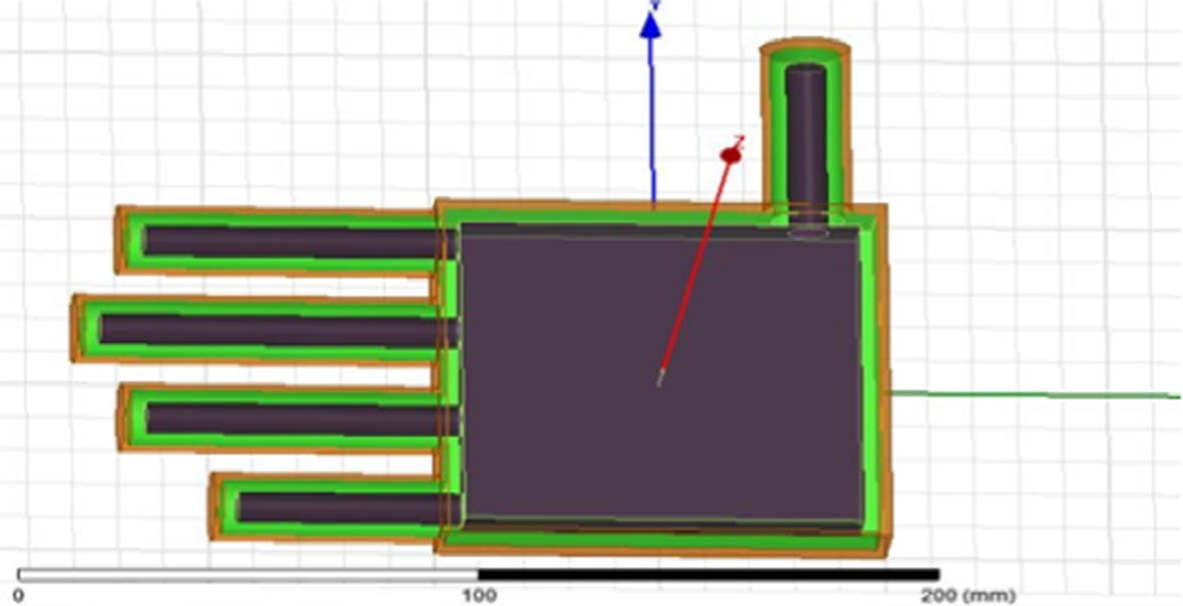
The bone layer in the hand model (shaded area). The human hand is a three-layer model that includes skin as the outer layer, solid materials as the second layer, and bones as the last layer

It is also designed on HFSS by using four cylinders of different lengths to represent the fingers and a box to represent the palm. The human hand is a three-layer model that contains skin as the outer layer, solid materials as the second layer, and bones as the last layer, as shown in Fig. 10 and Table 3. The human hand model layers solidity is taken in the form of 1000 kg/m^3^.

**Table 3:** Layers of a hand model and conductivity

Layers (in order)	Permittivity	Conductivity (siemens/m)
1. Skin	38.785	1.1847
2. Solid material	37.6	1.43
3. Bone	11.781	0.27522

## Results and discussion

In most BAN applications, we need a radiation pattern and an omnidirectional pattern, and the CPW antenna is an almost omnidirectional radiation pattern, which has proven that the PICA antenna is valuable for BANs applications.

**Fig. 11:**
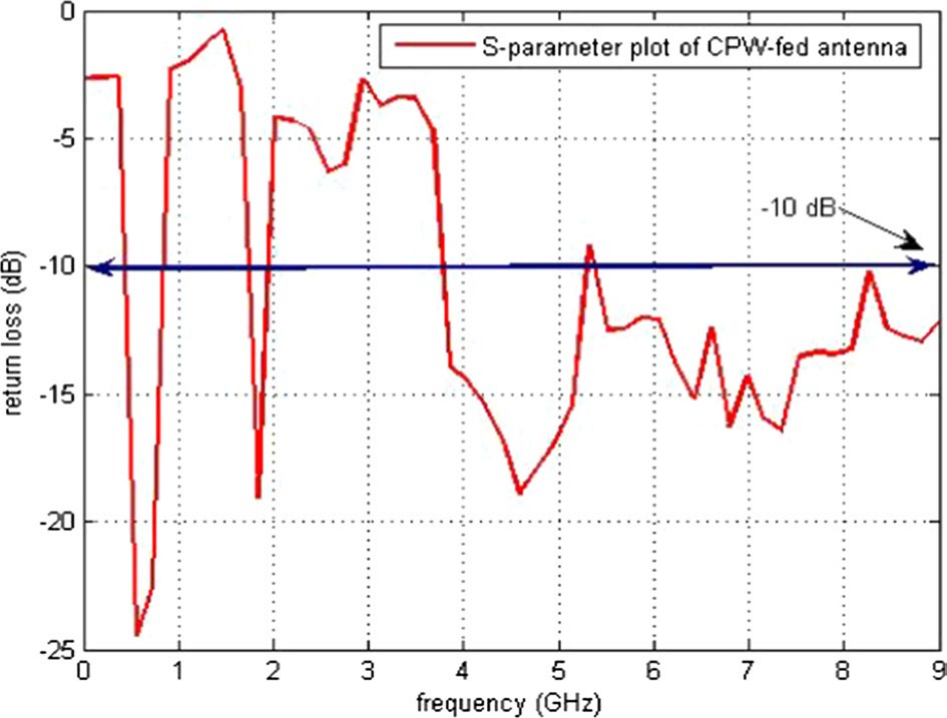
Return Loss versus Frequency. The return loss versus frequency is measured here, which indicates that at 0 frequency the return loss is − 3 and at 10 GHz frequency it is at − 12 dB

**Fig. 12:**
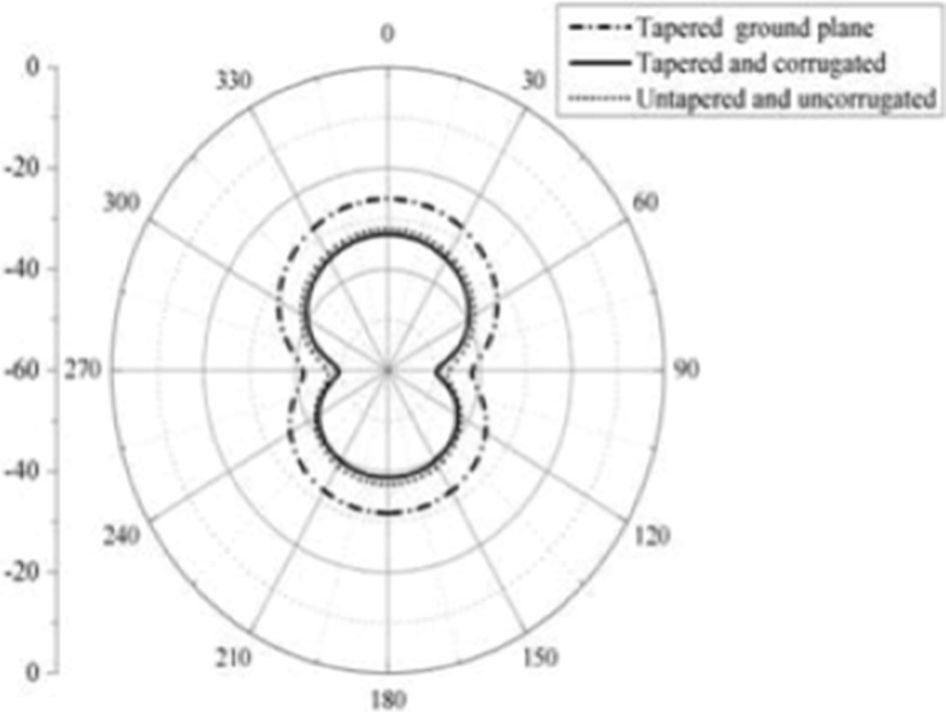
3D-polar plot. The 3D plot shows that it is evident from the 3D-polar plot of PICA antenna that the antenna has high gain which fulfills the requirement of the BANs applications

The return loss versus frequency graph is shown in Fig. 11. It is evident from the 3D polar plot of PICA antenna that the antenna has a high gain which fulfills the requirement of the BANs applications shown in Fig. 12.

### Wideband planar monopole antenna

The bandwidth of this antenna meets the requirements of mobile communications and UWB connections and is also valuable for the analysis of SAR results.

**Fig. 13:**
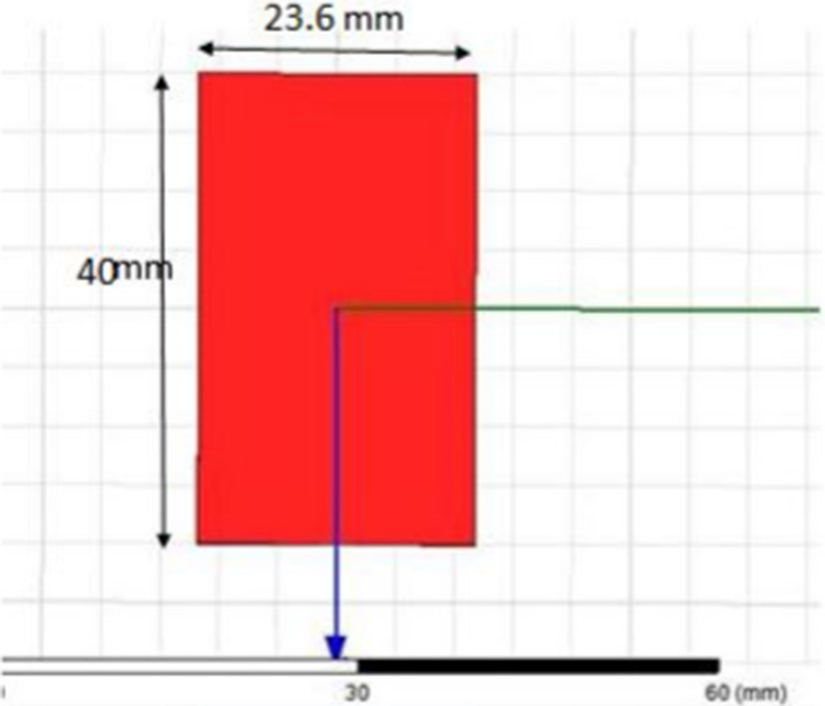
Ground plane (red color). In typical mobile communication systems, rapid changes have occurred; there is also technology change in handheld devices

In typical mobile communication systems, rapid changes have occurred, as shown in Fig. 13. There is also technology change in handheld devices.

The smaller dimensions and attractive design make the antenna space thinner and smaller, making it a difficult task for designers.

**Fig. 14:**
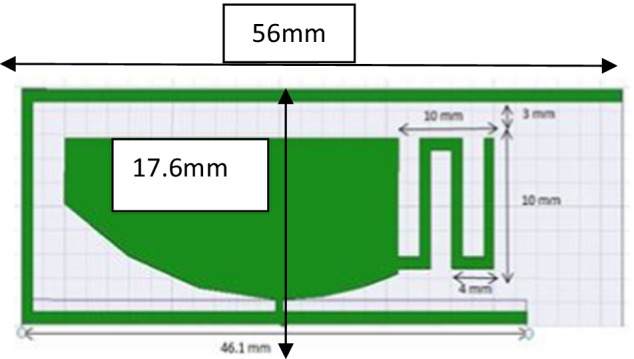
Radiating element. The base structure of the antenna consists of a lower layer of length equal to the wavelength of the fourth and the partial length of earth

The base structure of the antenna consists of a lower layer of length equal to the wavelength of the fourth and the partial length of the earth and the radioactive element with an inverted parasitic element of the L-strip. The antenna has a radiating element on the upper side as the antenna is dual band shown in Fig. 14.

**Fig. 15:**
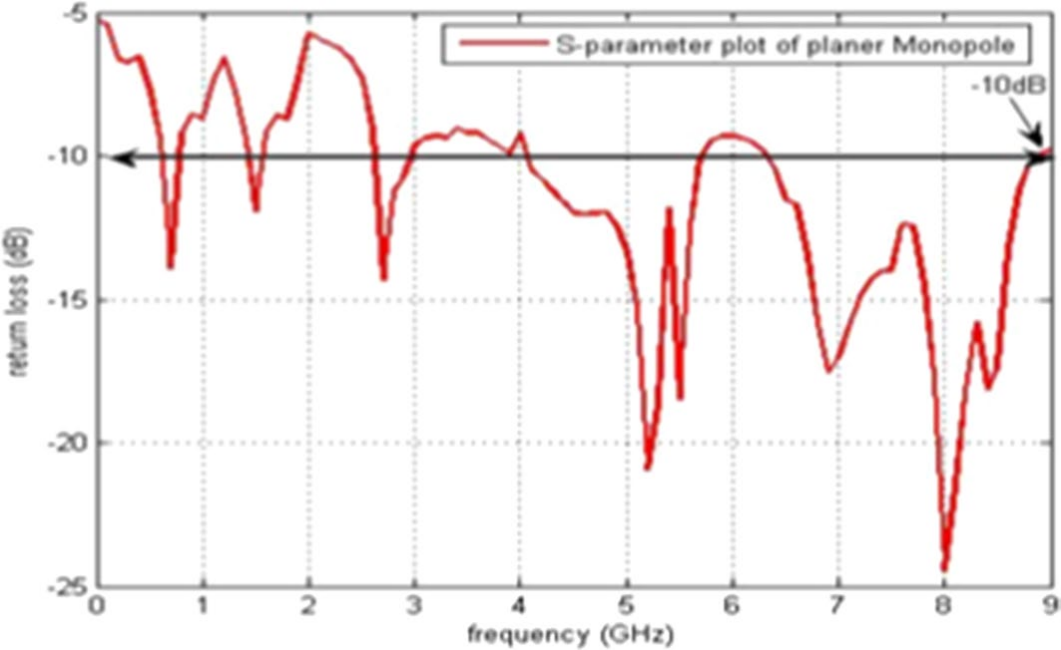
Return loss versus frequency graph. The return loss vs frequency is measured here, which indicates that at 0 frequency the return loss is -3 and at 10 GHz frequency it is at -10 dB

**Fig. 16:**
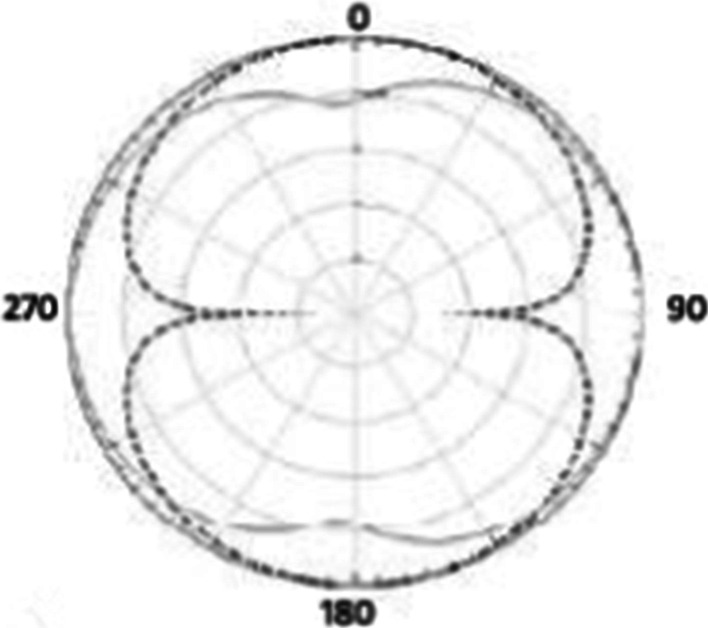
2D radiation pattern. The 2D radiation patterns are shown in the figure on 0, 90, 180, and 270 positions

Return loss versus frequency graph is shown in Fig. 15. Its radiating element comprises a chopped circular section and an inverted L-band interference element. Simulations and results of the 2D radiation pattern are shown in Fig. 16.

In BAN applications, a multidirectional radiation pattern is required and the radiation pattern of a monopole antenna is roughly an omnidirectional radiation pattern, indicating that the PICA antenna is useful for BAN applications. The antenna bandwidth is suitable for both UWB and mobile communications and for calculating the SAR result.

### Antenna manufacturing

**Fig. 17:**
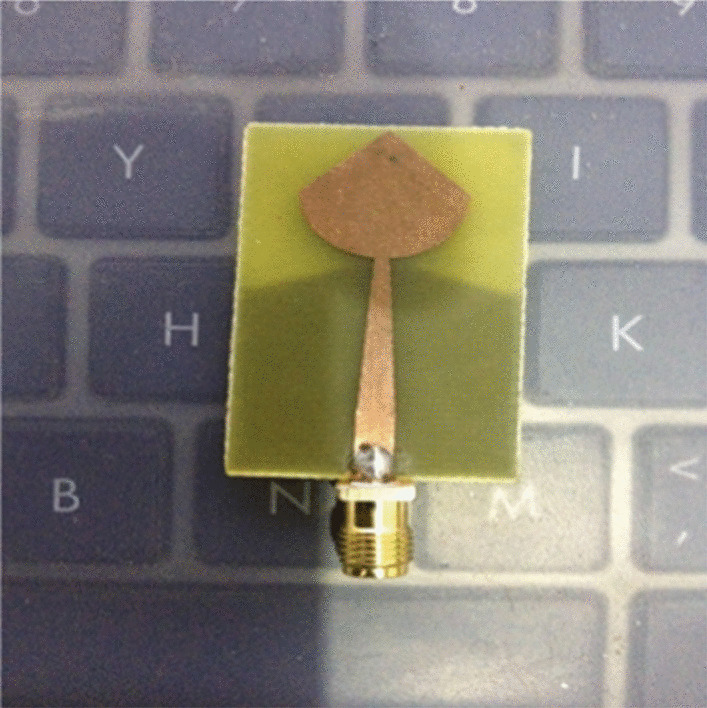
Fabricated PICA antenna. The return loss and radiation pattern various parameters are measured here

The above antennas are manufactured, and their various parameters are measured, such as return loss and radiation pattern, as shown in Fig. 17.

**Fig. 18:**
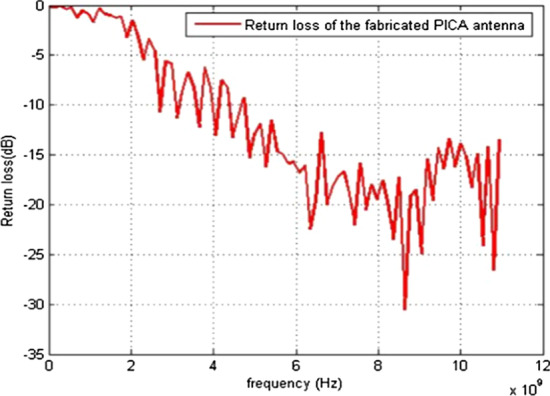
Return loss versus frequency of the fabricated PICA antenna. The PICA antenna is manufactured on an FR-4 (1.6 mm) blade, and an SMA connector is used for power

The PICA antenna is manufactured on an FR-4 (1.6 mm) blade, and an SMA connector is used for power, as shown in Fig. 18.

### Results of simulations of fabricated Antennas

**Fig. 19:**
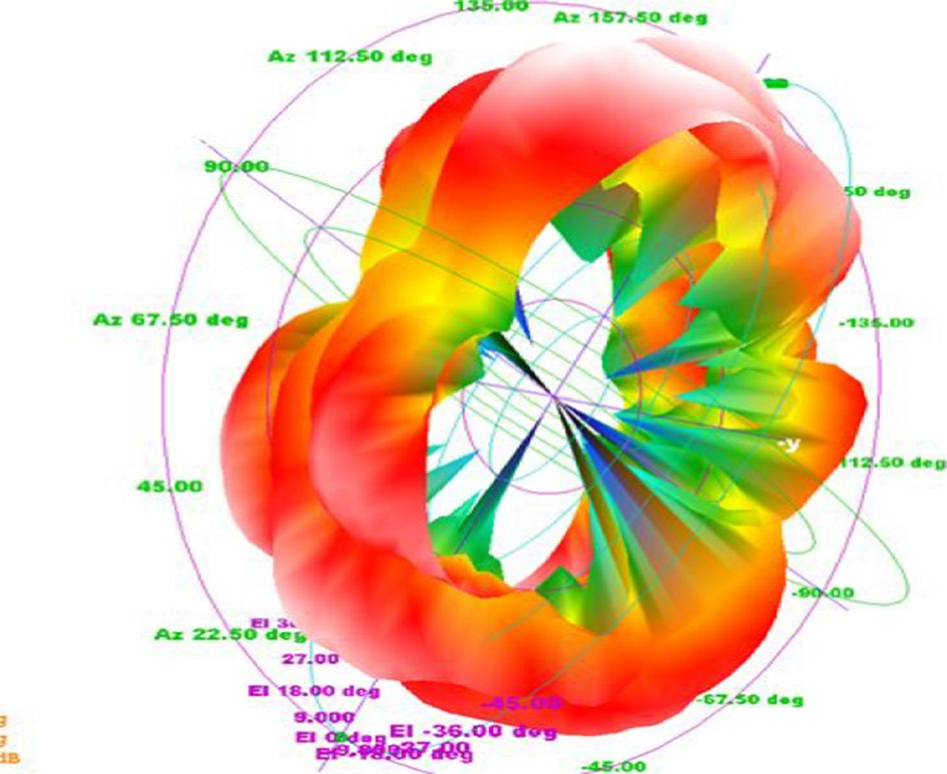
PICA antenna 3D radiation pattern. The radiation pattern of the manufactured product is almost omnidirectional, which is one of the critical requirements of the applications of the BANS that are presented here

**Fig. 20:**
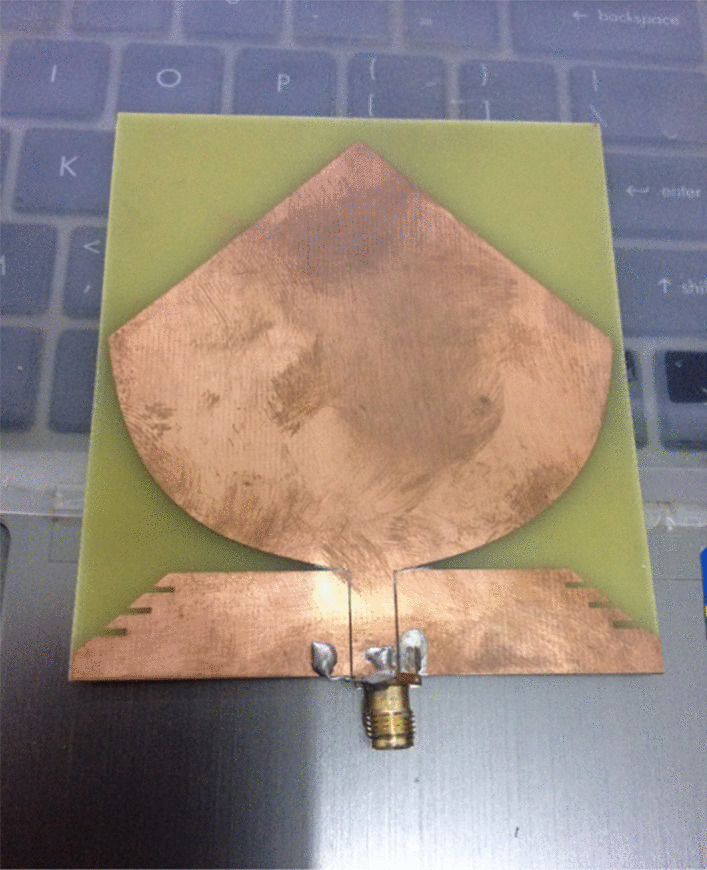
Fabricated CPW-fed planar inverted cone antenna is presented here. CPW-fed planar inverted cone antenna (PICA) for ultra-wideband (UWB) communication applications

The results show that the antenna bandwidth covers almost the entire UWB band and also has a loss of functional feedback of around − 30 dB. The radiation pattern of the manufactured product is almost omnidirectional, which is one of the critical requirements of BANS applications, as illustrated in Figs. 19 and 20.

**Fig. 21:**
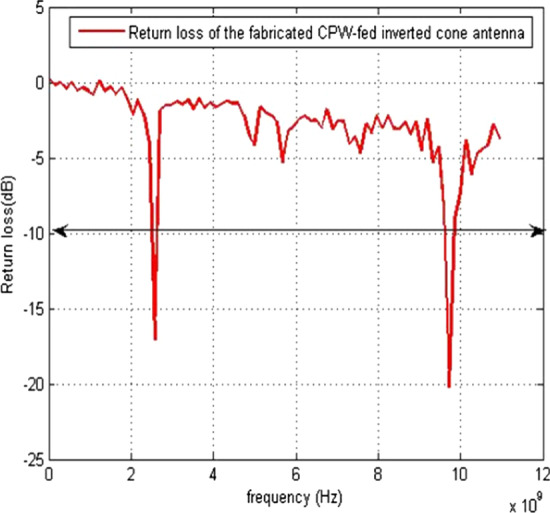
Return loss versus frequency of the fabricated PICA antenna. Fabricated PICA vs. frequency antennas and return loss responses of antennas when placed on the body

Figure 21 demonstrates the results of the fabricated antenna.

The results show that the bandwidth of the antenna is not large enough to cover the entire UWB band, for which we have changed the original substrate to the FR-4 sheet.

**Fig. 22:**
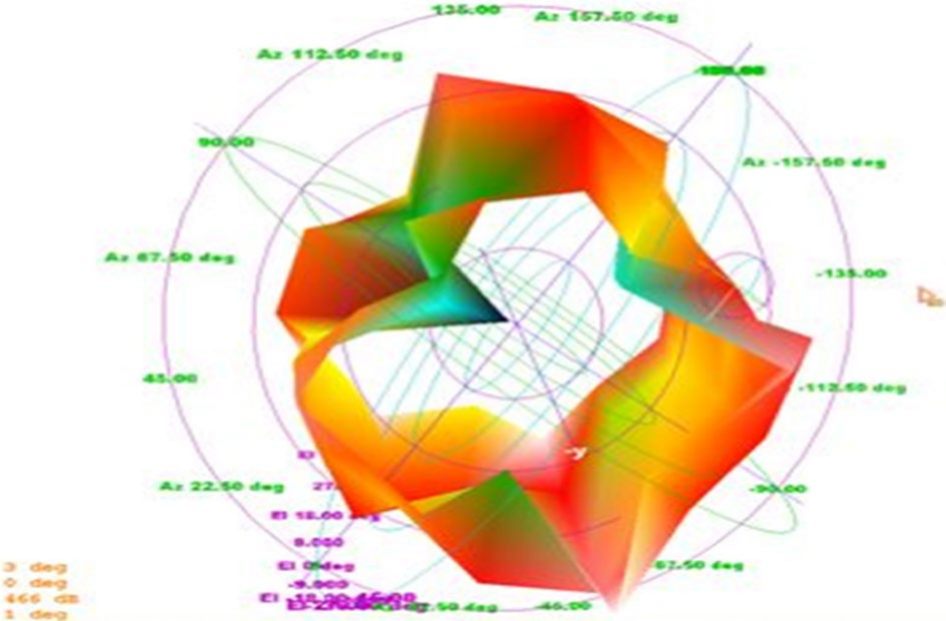
3D radiation pattern of the fabricated antenna. It is clear from the above plot that the radiation pattern of the fabricated is almost omnidirectional

**Fig. 23:**
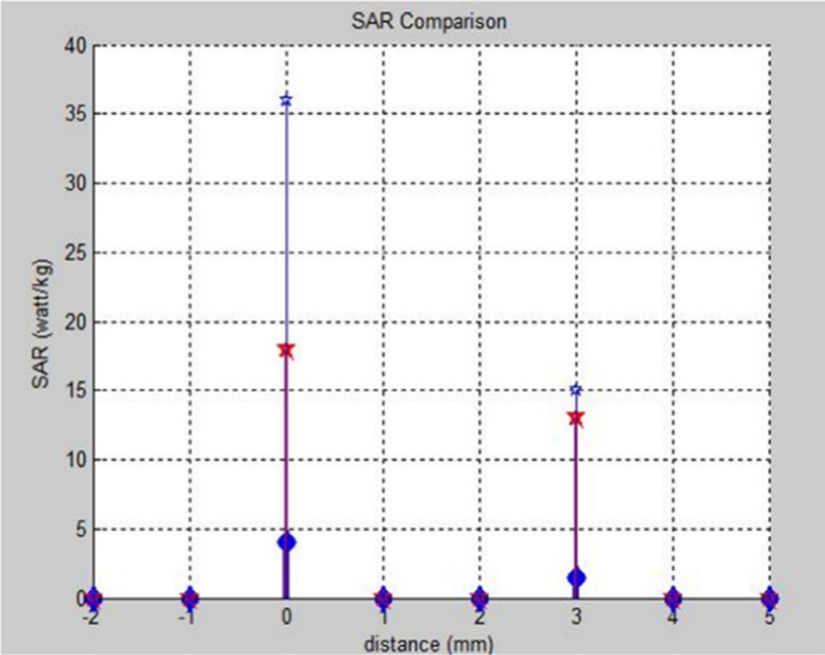
MATLAB plot of SAR values of antennas with a hand model, which illustrates the SAR comparison with the distance

It is clear from the above plot that the radiation pattern of the fabricated is almost omnidirectional. The results of the fabricated antennas are as desired, thus proving the antennas more valuable for the applications of BANs, as shown in Figs. 22 and 23.

### SAR analysis

The final phase of our project includes simulations of the antennas in the HFSS software with the head and hand models. We place the three antennas one by one on each model, that is to say the hand and the head, and study their SAR. Before starting the simulation, our main reason was to offer the best performing antenna in the SAR field. In addition, we will also analyze the strength of electromagnetic fields in different layers of designed models. The antennas are placed at three different distances from the hand and the head, namely 0 mm, 3 mm, and 5 mm.

#### PICA

The PICA antenna is placed with the three-layer hand model at three different distances, i.e., 0 mm, 3 mm, and 5 mm. The radiation limit is assigned to the far-field region. Simulations at a distance of 0 mm are explicitly designed to analyze SAR for area networks of the body because the antennas are placed mainly on the surface of the human body in BAN applications. The next two observation points are used to approximate the exact values. The local and average SAR values at three different distances obtained from simulations are shown in Table 4.

**Table 4:** Local and average SAR for PICA antenna with a hand model

The distance of the antenna from the hand model (mm)	Local SAR (watt/1 g)	Average SAR (watt/10 g)	SAR (watt/kg)
0	0.004	0.04	4
3	0.0015	0.015	1.5
5	0.0013	0.013	0.13

#### CPW-fed planar inverted cone antenna with hand model

The second simulation of hand is with CPW-fed inverted cone antenna. The radiation boundary is assigned based on the far-field region. The observation points for this antenna remain the same as the PICA antenna. Local and average SAR values at three different distances obtained as a result of simulations are given in Table 5. Local, average, and SAR CPW-fed inverted cone antenna models are calculated on the basis of 0 mm, 3 mm, and 5 mm.

**Table 5:** Local and average SAR for CPW-fed inverted cone antenna with a hand model

The distance of the antenna from the hand model (mm)	Local SAR (watt/1 g)	Average SAR (watt/10 g)	SAR (watt/kg)
0	0.018	0.18	18
3	0.013	0.13	13
5	0.07	0.7	7

#### Monopole antenna

Monopole antenna is simulated with a hand model in HFSS software. The radiation boundary assigned in this simulation is also on the basis of the far-field region. Local and average SAR values at three different distances obtained as a result of simulations are given in Table 6. Local, average, and SAR for monopole antenna model are calculated on the basis of 0 mm, 3 mm, and 5 mm.

**Table 6:** Local and average SAR for monopole antenna with a hand model

The distance of the antenna from the hand model (mm)	Local SAR (watt/1 g)	Average SAR (watt/10 g)	SAR (watt/kg)
0	0.036	0.36	36
3	0.015	0.15	15
5	0.011	0.11	11

#### Smart analysis with head model

Smart analysis of designed antennas was done by placing these different antennas on the designed head and hand model. SAR was calculated at these points.

*PICA* PICA antenna is placed with the head model at three different distances that are 0 mm, 3 mm, and 5 mm. The radiation boundary is assigned based on the far-field region. The next two observation points are taken to get nearer values to the accurate one. Local and average SAR values at three (i.e., 0 mm, 3 mm, and 5 mm) different distances obtained as a result of simulations are given in Table 7.

**Table 7:** Local and average SAR for PICA antenna with head model

Distance of the antenna from the hand model (mm)	Local SAR (watt/1 g)	Average SAR (watt/10 g)	SAR (watt/kg)
0	0.005	0.05	5
3	0.0019	0.019	1.9
5	0.0016	0.016	0.16

#### CPW-fed planar inverted cone antenna with head model

The following manual model simulation is performed with an inverted cone antenna powered by CPW. The observation points of this antenna remain the same as those of the PICA antenna. The local and average SAR values obtained at three different distances following simulations are given in Table 8.

**Table 8:** Local and average SAR for CPW-fed inverted cone antenna with the head model

Distance of the antenna from the hand model (mm)	Local SAR (watt/1 g)	Average SAR (watt/10 g)	SAR (watt/kg)
0	0.018	0.18	18
3	0.016	0.16	16
5	0.09	0.9	9

#### Monopole antenna

Local and average SAR values for monopole antenna at three different distances obtained as a result of simulations are given in Table 9.

**Table 9:** Local and average SAR for monopole antenna with a hand model

Distance of the antenna from the hand model (mm)	Local SAR (watt/1 g)	Average SAR (watt/10 g)	SAR (watt/kg)
0	0.023	0.23	23
3	0.014	0.14	14
5	0.08	0.8	8

**Fig. 24:**
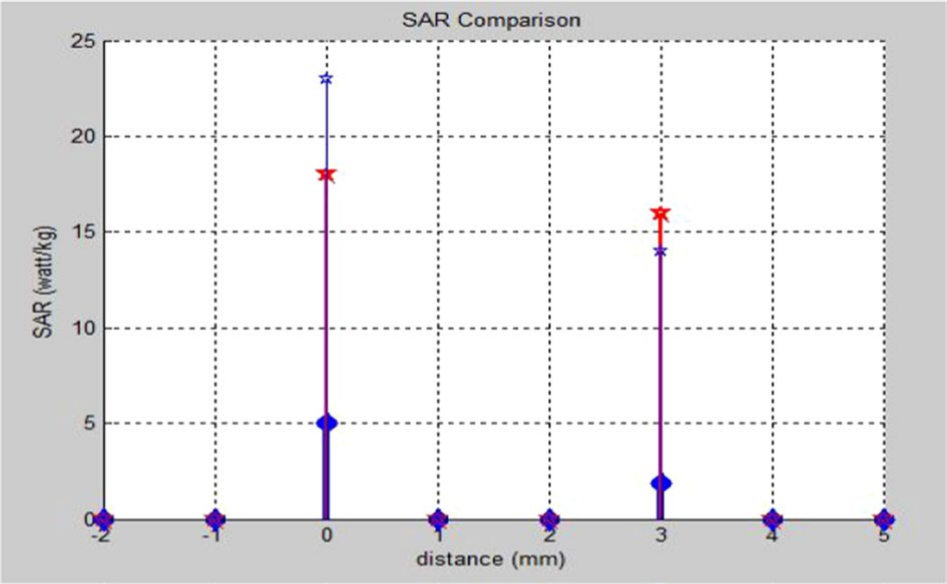
MATLAB plot of SAR values of antennas with a hand model, which shows the SAR comparison with the distance

The comparison of the SAR values for all three antennas is plotted in MATLAB, as shown in Fig. 24.

From the intelligent analysis of the simulations of the three antennas, we conclude that the SAR of the PICA antenna is better (lower) than the broadband inverted cone antenna and the CPW. As the distance between the antenna and the head and hand models increases, the SAR curve decreases almost exponentially. Multilayer fabrics are superior to the monolayer model in terms of accuracy of results, that is, the penetration of changes in electromagnetic fields, layers with different dielectric properties. Electromagnetic fields have a significant effect on the outer layers, that is, on the skin, and have the least effect on the inner layers, such as the CSF and the brain. The designed antennas are designed to be portable and lightweight for intelligent applications.

## Conclusion

UWB technology has more advantages than narrowband when considering BAN applications. The bandwidth and the omnidirectional radiation pattern show that the three antennas are useful for BANs. Apart from that, the designed antennas also have other advantages, such as low cost, simplicity of structure, and lightness. The design of the proposed antennas makes smart apps and health monitoring systems supportive. SAR is the most useful parameter for calculating the rate of energy absorption by human tissue. Human head and hand single-layer models are poor models compared to multilayer models in terms of accuracy of SAR results. The SARs are higher for high permittivity layers. The intelligent analysis of these simulations concluded that of the three designs of PICA antennas, CPW-fed inverted cone antenna, and broadband monopolar antenna. The PICA antenna is better than SAR. The PICA antenna has the lowest risk of damaging human tissue compared to the other two antennas. In addition, the PICA antenna applies to UWB and mobile communications because the antenna SAR level is below the limits set by the FCC.

In the future, we aim to design the multilayers of human phantoms in order to get real-time values of SAR and then compare it to the simulated values. The other human body parts can be used to improve the results. We aim to take the real-time values of fabricated antennas. The fabrication of antennas is completed, and their various results are measured. This work will then prove the designed antennas favorable for the number of applications of BANs.

## Data Availability

No data and materials are required.

## Abbreviations

BAN: Body area network
UWB: Ultra-wide band
SAR: Specific absorption rate
HFSS: High-frequency structure simulator
RF: Radio frequency
EM: Electromagnetic
FSS: Fixed satellite service
IoT: Internet of things
RDF: Radio direction finder
SPARQL: Simple Protocol and RDF Query Language
WBAN: Wireless body area network
PICA: Planar inverted cone antenna
CPW: Coplanar waveguide
SMA: Sub-miniature version A
CSF: Cerebrospinal fluid
CPW: Coplanar waveguide
FCC: Federal Communications Commission
